# Polarized nature of the COVID-19 pandemic in Japan: associations with population age structure and behaviours

**DOI:** 10.1186/s41182-021-00324-0

**Published:** 2021-05-13

**Authors:** Junko Okumura

**Affiliations:** 1 Institute of Tropical Medicine, Nagasaki, Japan; 2 School of Tropical Medicine and Global Health, Nagasaki University, 1-12-4 Sakamoto, Nagasaki, Nagasaki 852-8523 Japan

**Keywords:** Behaviour, COVID-19, Japan, Perception, Population age structure

## Abstract

**Background:**

Although the scale of the coronavirus disease (COVID-19) pandemic was relatively small in Japan compared with the rest of the world, the polarisation of areas into high- and low-COVID-19-incidence areas was observed among the 47 prefectures. The aims of this study were not only identifying the factors associated with the polarised COVID-19 pandemic in Japan but also discussing effective preventive measures.

**Methods:**

This was an ecological study using online survey data which was cross-sectionally conducted by the author. A total of 6000 respondents who resided in 10 low- and 10 high-COVID-19 incidence prefectures, with a wide gap in terms of COVID-19 incidence, in Japan were recruited. Data on COVID-19 cases and geodemographic information were obtained from official government sites. Statistical analyses were conducted to compare variables between the two areas and age groups.

**Results:**

This study revealed that that age influenced people’s behaviours and perceptions, except one behaviour of ‘wearing facemasks’. The major factors significantly associated with the cumulative number of COVID-19 cases per 100,000 people were ‘commuting by private automobile’ (adjusted odds ratio [AOR], 0.444; 95% confidence interval [CI], 0.394–0.501), ‘commuting by public transportation’ (AOR, 6.813; 95% CI, 5.567–8.336), ‘washing hands’ (AOR, 1.233; 95% CI, 1.005–1.511), ‘opening windows regularly’ (AOR, 1.248; 95% CI, 1.104–1.412), ‘avoiding crowded places (AOR, 0.757; 95% CI, 0.641–0.893), ‘non-scheduled visits to drinking places’ (AOR, 1.212; 95% CI, 1.054–1.392) and ‘perceived risk of contracting COVID-19’ (AOR, 1.380; 95% CI, 1.180–1.612). These factors were strongly associated with age groups.

**Conclusions:**

Effective preventive measures for COVID-19 transmission can be developed by understanding the characteristics of populated areas, such as public transportation infrastructure and younger people’s movements and behaviours in relation to the population age structure to contain the current epidemic and protect the most vulnerable elderly people.

## Background

In December 2019, coronavirus disease (COVID-19) emerged in Wuhan city, located in Hubei Province, the People’s Republic of China [1]. In Japan, the first case of COVID-19 was reported on 16 January 2020 [2]. The number of reported cases increased gradually, reaching 4111 cases on 6 April 2020. To control the COVID-19 outbreak, the government of Japan declared a state of emergency in seven prefectures on 7 April 2020 and extended it to all 47 prefectures on 16 April 2020 [3, 4].

Although, the scale of the COVID-19 epidemic was relatively small in Japan, compared with that in the rest of the world [5], the polarisation of areas into high- and low-COVID-19-incidence areas were observed among the 47 prefectures. On 6 April 2020, the cumulative number of reported cases in Iwate and Tottori prefectures were zero, while those in the Tokyo metropolis and Osaka prefecture were 1130 and 428, respectively. By 15 April 2020, the numbers were zero and one in Iwate and Tottori prefectures, respectively; however, those in Tokyo and Osaka have doubled [6]. The reported deaths attributed from COVID-19 were relatively small in Japan compared to the USA; as of 6 April 2020, the cumulative number of deaths in Japan and the USA were 97 and 8358, respectively [6, 7].

The epidemiological data monitored continually raised multiple questions. Was the polarisation due to differences in population density? Were there any other factors that widened the gap? Did Japanese cultural factors, such as people’s awareness on issues related to COVID-19 and subsequent changes adopted in daily practices, influence the incidence of COVID-19? In addition, how does the age structure of the population influence people’s preventive behaviours and perceptions, widening the gap in COVID-19-incidence rates between the bottom 10 (low-incidence areas) and top 10 (high-incidence areas) prefectures? To answer these questions, this study aimed to identify the possible factors associated with the incidence gap between low- and high-incidence areas to establish more effective advocacy measures for managing COVID-19, considering the Japanese population’s age structure.

## Methods

### Recruitment of the respondents

This was an ecological study using online survey data of behaviours and perceptions which was cross-sectionally conducted by the author. The respondents were recruited from Rakuten Insight, an online research company in Japan with 2.3 million research respondents comprising a survey panel. The respondents generally receive a certain amount of Rakuten points (equivalent to a few United States dollars), which can be availed for online shopping at the Rakuten shopping site. According to a ranking of cumulative incidence reported daily by the Nihon Hoso Kyokai [6], the bottom and top 10 prefectures, as of 23 April 2020, were selected from the 47 prefectures in Japan. Before the survey began, it was anticipated that the number of COVID-19 cases might rapidly increase even in areas with zero reported cases. Therefore, these areas were also included. However, at the end of the survey, one prefecture still had zero reported cases of COVID-19.

The author then requested that the online survey site be accessible until the number of respondents living in each prefecture reached 300. The sample size was not decided using population-based proportionate sampling; rather, it was decided with intention to maximise the number of participating individuals aged ≥ 60 years and 18–29 years. Based on the respondent’s age, they were categorised into six age categories: 18–29, 30–39, 40–49, 50–59, 60–69 and ≥ 70 years old.

### Data collection and variables

**Fig. 1:**
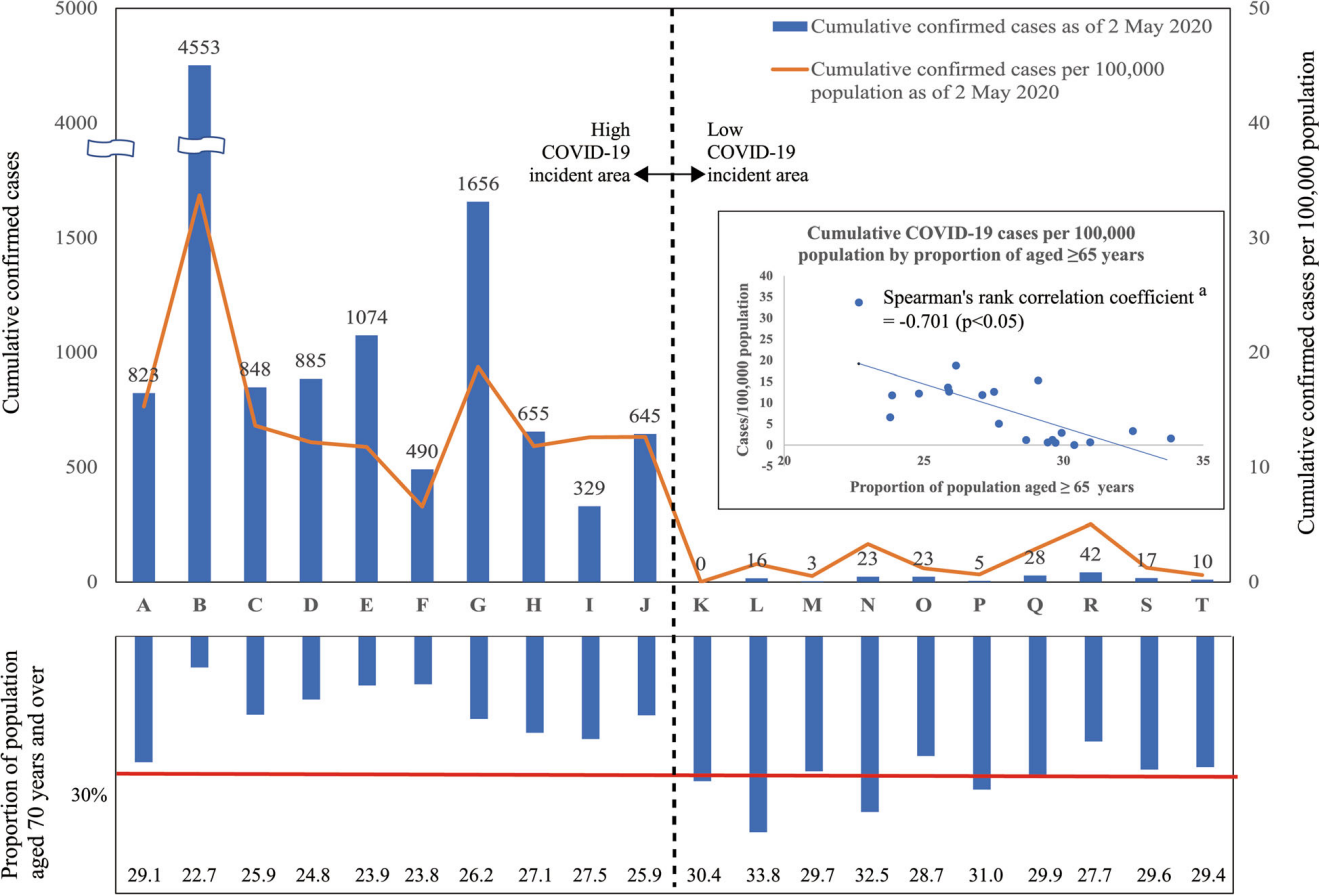
Cumulative COVID-19 cases, cases per 100,000 population and proportion of population aged ≥ 65 years. ^a^ Due to non-normal distribution of data, Spearman’s rank correlation test was adopted. Cumulative COVID-19 cases and cases per 100,000 population are calculated based on reported cases as of 2 May 2020

An original questionnaire was developed with advocacy messages provided by the Japanese government [3, 4, 8, 9]. The study variables included the town of residence; socio-demographic characteristics (age, occupation, number of household members by age category), knowledge of COVID-19 (symptoms, preventive measures, source of information), lifestyle (means of commuting, places regularly visited, travel destinations) and perceived risks. The online survey site was accessible to eligible respondents from 1 May to 6 May 2020. However, the number of respondents in the 20 target prefectures reached the target by midnight of 1 May 2020. Based on the cumulative number of COVID-19 cases per 100,000 people on 2 May 2020 (hereafter referred to as CASES per 100,000 on 02/05/2020), the study areas were sub-divided into low (bottom 10) and high (top 10) incidence areas. There was no difference between the sub-divided areas and the division of the original area of the survey panel (Fig. 1). In addition, data published online, such as the cumulative number of reported COVID-19 cases on each index date [6], proportions of age-specific population by sex and population density in each target prefecture, were obtained from the official statistical data of Japan [10].

### Data analyses

Spearman’s rank correlation coefficient was used to test correlations among ‘population density’, ‘proportion of population aged 65 years and over’ and ‘cases per 100,000 population as of 2 May 2020’ by using official statistics [10]. All the variables collected from respondents were weighted with population by sex and age of each target area [10] when the statistical tests were conducted. Pearson’s chi-squared (χ^2^) test, Student’s *t* test and Mann-Whitney *U* test were used to compare variables between the groups (areas with low- and high-COVID-19-incidence, and age group of 18–59 years and ≥ 60 years). Univariate binomial analyses were conducted to test associations of behaviours and perceptions with age group. Univariate and multivariate binomial analyses were conducted to identify areas with associated factors for high COVID-19 incidence per 100,000 on 02/05/2020. Odds ratios (ORs) and adjusted odds ratios (AORs) were then derived with a 95% confidence interval (CI). The level of significance was set at *p* < 0.05. Data were analysed using SPSS software version 22.0 for Windows (IBM Corp., Armonk, NY, USA).

### Ethics

The study was approved by the Ethical Review Board of the Institute of Tropical Medicine, Nagasaki University (authorization number of 200409235). Each respondent was informed about this study, and they had all the rights and means to cancel their entry. The respondents who provided consent to respond to the questions clicked the ‘Continue’ button to proceed with the process. Only the responses of those who completed the self-administered questionnaire were uploaded as survey data. Anonymized data were provided by Rakuten Insight for these analyses.

## Results

### Correlations between demographic data and COVID-19 incidence

**Fig. 2:**
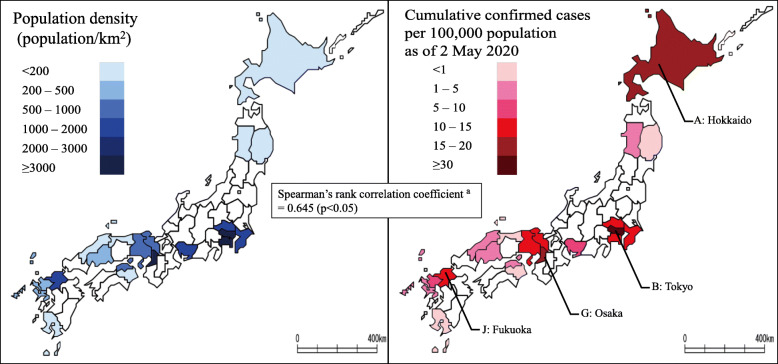
Population density and cumulative COVID-19 cases per 100,000 population in the study areas. ^a^ Due to non-normal distribution of data, Spearman’s rank correlation test was adopted. Cumulative COVID-19 cases and cases per 100,000 population are calculated based on reported cases as of 2 May 2020

CASES per 100,000 on 02/05/2020 in the low- and high-incidence areas were 1.2 (interquartile range [IQR] 2.4) and 12.6 (IQR 4.3), respectively. Additionally, CASES per 100,000 on 02/05/2020 was negatively correlated with the proportion of the population aged ≥ 65 years (the Spearman’s rank correlation coefficient, − 0.701; *p* = 0.001). Population density was lower in the low-incidence area (174.9 population/km^2^; IQR 227.9 population/km^2^) than in the high-area (1338.7 population/km^2^; IQR 3385.6 population/km^2^). Population density was associated with the number of cases per 100,000 people, except in some areas such as Hokkaido (prefecture A). The Spearman’s rank correlation coefficient was 0.645 (*p* < 0.001) (Fig. 1, Fig. 2, Table 1).

**Table 1:** Geographical and demographical characteristics of the study area

	Low-COVID-19-incidence area, *n* = 10, Median (IQR, Min–Max)	High-COVID-19-incidence area, *n* = 10, Median (IQR, Min–Max)
Population density (population/km^2^) [10]	174.9 (227.9, 80.3–509.4)	1338.7 (3385.6), (66.9–6354.8)
Cumulative COVID-19 cases per 100,000 population as of 2 May 2020 [6]	1.2 (2.4, 0–5.0)	12.6 (4.3, 6.6–33.7)
Proportion (%) of population aged 65 years [10]	30.0 (2.1, 27.7–33.8)	25.9 (3.4, 22.7–29.1)

*IQR* interquartile range, *Min* minimum, *Max* maximum

### Characteristics of respondents and areas

Altogether, responses of 6000 respondents were recorded with representation from 20 prefectures in Japan. Table 2 provides both unweighted and weighted characteristics. There was no difference in the sex ratio between the low- and high-incidence areas (*p* = 0.301). In low- and high-incidence areas, the mean of the household size were 3.2 persons/household (95% CI 3.2–3.3) and 3.0 persons/household (95% CI 3.0–3.1) respectively (*p* < 0.001); and the mean of the number of family members aged ≥ 60 years in low- and high-incidence areas were 1.3 persons (95% CI 1.2–1.3) and 1.1 (95% CI 1.0–1.1) respectively (*p* < 0.001). Employment status varied between the two areas (*p* < 0.001), and the proportion of people out-of-work in the low-incidence area was 21% while in the high-incidence area was 17%. Moreover, the median for respondent age in the low-incidence area was 55 years old which was older than the high-incidence area (median 51 years) (*p* < 0.001).

**Table 2:** Characteristics of respondents by study area (un-weighted and weighted)

	Un-weighed		Weighted		*p* value^a^
	Low-COVID-19-incidence area, *n* = 3000	High-COVID-19-incidence area, *n* = 3000	Low-COVID-19-incidence area, *n* = 3000	High-COVID-19-incidence area, *n* = 3000	
Sex					
Female (*n*, %)	1363 (45%)	1337 (45%)	1597 (53%)	1556 (52%)	0.301
Male (*n*, %)	1637 (55%)	1663 (55%)	1403 (47%)	1444 (48%)	
Household size (mean, 95% CI)	3.3 (3.3–3.4)	3.0 (3.0–3.1)	3.2 (3.2–3.3)	3.0 (3.0–3.1)	< 0.001
Number of family member ≥ 60 years old	1.1 (1.0–1.1)	0.9 (0.9–1.0)	1.3 (1.2–1.3)	1.1 (1.0–1.1)	< 0.001
Employment status (*n*, %)					
Employed	1574 (53%)	1524 (51%)	1215 (41%)	1329 (44%)	< 0.001
Unpaid (homemaker, eldercare, etc.)	284 (10%)	288 (10%)	552 (18%)	509 (17%)	
Self-employed	121 (4%)	108 (4%)	103 (3%)	93 (3%)	
Part-time job	393 (13%)	428 (14%)	437 (15%)	475 (16%)	
Student	119 (4%)	163 (5%)	119 (4%)	73 (2%)	
Out-of-work (including retired)	509 (17%)	489 (16%)	643 (21%)	520 (17%)	
Work in healthcare setting (n, %)	281(9%)	169 (6%)	193 (6%)	139 (5%)	0.003
Commuting (*n*, %)	2094 (50%)	2057 (50%)	1710 (48%)	1837 (52%)	0.001
Age (median), (IQR, Min–Max))	45 (34, 18–87)	47 (34, 18–89)	55 (31, 18–87)	51 (31, 18–89)	< 0.001

*IQR* interquartile range, *Min* minimum, *Max* maximum

^a^*p* value is based on the Chi-squared test, *t* test and Mann-Whitney *U* test

### Behaviours and perceptions by age categories

**Fig. 3:**
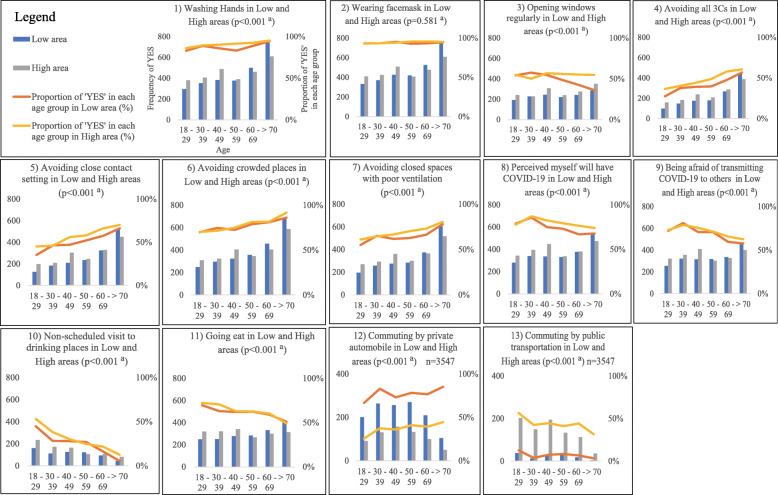
(1–13) Behaviours and perceptions between low and high COVID-19 incidence areas by six age groups. Low: Low-COVID-19-incidence-area; High: High-COVID-19-incidence-area. All the graphs are based on weighted figures; from (1) to (11), the sample size: 6000 respondents; (12) and (13), the sample size is based on commuting respondents: 3547 respondents. ^a^*p* value is based on the chi-squared test

In both low- and high-incidence areas, more than 94% of people wore facemasks, and there was no difference in the rate of ‘wearing facemasks’ by age group (*p* = 0.631 and *p* = 0.301, respectively). All the other variables (behaviours and perceptions) stratified by age categories, depicted in (Fig 3(1–13)), were significantly different between low- and high-incidence areas (*p* < 0.001).

When the behaviours and perceptions were compared by age group, ‘younger age group (aged 18–59 years)’ and ‘older age group (aged ≥ 60 years)’, the respondents in older age group adhered to preventive practice as compared to the other group. The odds ratios of ‘avoiding closed spaces with poor ventilation’, ‘avoiding crowded places’and ‘avoiding close contact setting’ were 1.719 (95% CI 1.534–1.926), 2.308 (95% CI 1.781–2.333), and 1.961 (95% CI 1.764–2.180) respectively. Regarding ‘washing hands’, respondents in the older age group were more likely to adhere to this practice than the other group (OR 1.914; 95% CI 1.592–2.300). The major practices and perceptions that older age group responded as ‘NO’ were ‘ commuting by public transportation’ (OR 0.257; 95% CI 0.216–0.306), ‘opening windows regularly’ (OR 0.732; 95% CI 0.661–0.812), ‘non-scheduled visit to drinking places’ (OR 0.291; 95% CI 0.254–0.333), ‘perceived risk of contracting COVID-19’ (OR 0.615; 95% CI 0.546–0.693) and ‘being afraid of transmitting COVID-19 to others’ (OR 0.543; 95% CI 0.486–0.607) (Table 3).

**Table 3:** Associations between behaviours/perceptions and age groups

Respondents’ behaviours and perceptions		Weighted *n* = 6000	
		Numbers (%) of respondents aged 18–59 years *n* = 3514	Numbers (%) of respondents aged ≥ 60 years *n* = 2487
Commuting by private automobile	No	2011 (57%)	2023 (81%)
	Yes	1503 (43%)	464 (19%)
	OR (95% CI)^a^	ref	0.307 (0.272–0.346)***
Commuting by public transportation	No	2729 (78%)	2316 (93%)
	Yes	784 (22%)	171 (7%)
	OR (95% CI)	ref	0.257 (0.216–0.306)***
Washing hands	No	440 (13%)	173 (7%)
	Yes	3074 (88%)	2313 (93%)
	OR (95% CI)	ref	1.914 (1.592–2.300)***
Practicing cough etiquette	No	1029 (29%)	922 (37%)
	Yes	2484 (71%)	1565 (63%)
	OR (95% CI)	ref	0.703 (0.630–0.784)***
Opening windows regularly	No	1614 (46%)	1335 (54%)
	Yes	1900 (54%)	1151 (46%)
	OR (95% CI)	ref	0.732 (0.661–0.812)***
Avoiding all 3 Cs^b^	No	2121 (60%)	1097 (44%)
	Yes	1393 (40%)	1389 (56%)
	OR (95% CI)	ref	1.928 (1.737–2.139)***
Avoiding closed spaces with poor ventilation	No	1279 (36%)	621 (25%)
	Yes	2235 (64%)	1865 (75%)
	OR (95% CI)	ref	1.719 (1.534–1.926)***
Avoiding crowded places	No	901 (26%)	360 (15%)
	Yes	2612 (74%)	2127 (85%)
	OR (95% CI)	ref	2.038 (1.781–2.333)***
Avoiding close-contact setting	No	1798 (51%)	866 (35%)
	Yes	1715 (49%)	1620 (65%)
	OR (95% CI)	ref	1.961 (1.764–2.180)***
Non-scheduled visit to drinking places	No	2317 (66%)	2162 (87%)
	Yes	1197 (34%)	325 (13%)
	OR (95% CI)	ref	0.291 (0.254–0.333)***
Going to eat	No	1208 (34%)	1139 (46%)
	Yes	2306 (66%)	1348 (54%)
	OR (95% CI)	ref	0.620 (0.558–0.689)***
Perceived risk of contracting COVID-19	No	708 (20%)	723 (29%)
	Yes	2806 (80%)	1763 (71%)
	OR (95% CI)	ref	0.615 (0.546–0.693)***
Being afraid of transmitting COVID-19 to others	No	909 (26%)	973 (39%)
	Yes	2604 (74%)	1514 (61%)
	OR (95% CI)	ref	0.543 (0.486–0.607)***
Wearing facemask	No	217 (6%)	134 (5%)
	Yes	3296 (94%)	2352 (95%)
	OR (95% CI)	ref	1.156 (0.926–1.442)

***p < 0.001

^a^Univariate binomial analyses were conducted to calculate odds ratios (ORs) (95% confidence interval [CI])

^b^‘Avoiding all 3 Cs’ meaning that avoiding ‘closed spaces with poor ventilation’, ‘crowded places’ and ‘close contact setting’, such as close-range conversations

### Factors associated with COVID-19 incidence

Table 4 depicts the major factors associated with ‘CASES per 100,000 on 02/05/2020’ in the low- and high-incidence areas. The univariate binominal analyses indicated that all variables, except ‘avoiding crowded places’ and ‘wearing a facemask’, were significantly associated with CASES per 100,000 on 02/05/2020, and they contributed to the difference in incidence. After adjusting for all variables, except ‘avoiding the 3 Cs (Cs: closed spaces with poor ventilation, crowded places and close contact setting, such as close-range conversations),’ the variables that showed significant positive associations with high-incidence area were ‘commuting by public transportation, such as trains, subways and buses’ (AOR, 6.813; 95% CI, 5.567–8.336), ‘washing hands’ (AOR, 1.233; 95% CI, 1.005–1.511), ‘opening windows regularly’ (AOR, 1.248; 95% CI, 1.104–1.412), ‘non-scheduled visit to drinking places’ (AOR, 1.212; 95% CI, 1.054–1.392) and ‘perceived risk of contracting COVID-19’ (AOR, 1.380; 95% CI, 1.180–1.612). The factors that were significantly negatively associated with high-incidence areas were ‘commuting by private automobile’ (AOR, 0.444; 95% CI, 0.394–0.501) and ‘avoiding crowded places’ (AOR, 0.757; 95% CI, 0.641–0.839).

**Table 4:** Univariate and multivariate binomial analyses of factors associated with high COVID-19 incidence area

Respondents’ behaviours and perceptions		Un-weighted, *n* = 6000		Weighted, *n* = 6000			
		Numbers (%) in low-COVID-19-incidence area	Numbers (%) in high-COVID-19-incidence area	Numbers (%) in low-COVID-19-incidence area	Numbers (%) in high-COVID-19-incidence area	Univariate binominal analysis for high-COVID-19 incidence area OR (95% CI)	Multivariate binominal analysis for high-COVID-19 incidence area AOR (95% CI)
Commuting by private automobile	No	1447 (48%)	2294 (77%)	1696 (56%)	2337 (78%)	**ref**	**ref**
	Yes	1553 (52%)	706 (24%)	1304 (44%)	663 (22%)	0.369 (0.330–0.413)***	0.444 (0.39–0.501)***
Commuting by public transportation	No	2809 (94%)	2008 (67%)	2874 (96%)	2172 (72%)	**ref**	**ref**
	Yes	191 (6%)	992 (33%)	126 (4%)	828 (28%)	8.695 (7.151–10.573)***	6.813 (5.567–8.336)***
Washing hands	No	421 (14%)	307 (10%)	350 (12%)	264 (9%)	ref	ref
	Yes	2579 (86%)	2693 (90%)	2650 (88%)	2736 (91%)	1.369(1.157–1.620)***	1.233 (1.005–1.511)*
Practicing cough etiquette	No	988 (33%)	922 (31%)	1039 (35%)	912 (30%)	ref	ref
	Yes	2012 (67%)	2078 (69%)	1961 (65%)	2088 (70%)	1.213 (1.089–1.352)**	1.014 (0.890–1.155)
Opening windows regularly	No	1541 (51%)	1409 (47%)	1590 (53%)	1359 (45%)	**ref**	**ref**
	Yes	1459 (49%)	1519 (53%)	1410 (47%)	1641 (55%)	1.362 (1.230–1.507)***	1.248 (1.104–1.412)***
Avoiding all 3 Cs^a^	No	1888 (63%)	1636 (55%)	1685 (56%)	1533 (51%)	ref	–^b^
	Yes	1112 (37%)	1364 (45%)	1315 (44%)	1467 (49%)	1.226 (1.108–1.357)***	
Avoiding closed spaces with poor ventilation	No	1189 (40%)	988 (33%)	1001 (33%)	899 (30%)	ref	ref
	Yes	1811 (60%)	2012 (67%)	1999 (67%)	2101 (70%)	1.170 (1.050–1.305)**	1.059 (0.912–1.229)
Avoiding crowded places	No	742 (25%)	702 (23%)	641 (21%)	620 (21%)	ref	ref
	Yes	2258 (75%)	2298 (77%)	2359 (79%)	2380 (79%)	1.043 (0.921–1.181)	0.757 (0.641–0.893)**
Avoiding close-contact setting	No	1604 (54%)	1355 (45%)	1401 (47%)	1264 (42%)	ref	ref
	Yes	1396 (47%)	1645 (55%)	1599 (53%)	1736 (58%)	1.203 (1.087–1.333)***	1.106 (0.972–1.259)
Non-scheduled visit to drinking places	No	2098 (70%)	1927 (64%)	2344 (78%)	2135 (71%)	**ref**	**ref**
	Yes	902 (30%)	1073 (36%)	656 (22%)	865 (29%)	1.448 (1.288–1.628)***	1.212 (1.054–1.392)*
Going eat	No	1086 (36%)	1028 (34%)	1209 40%)	1138 (38%)	ref	ref
	Yes	1914 (64%)	1972 (66%)	1791 (60%)	1862 (62%)	1.105 (0.996–1.225)	0.899 (0.798–1.014)
Perceived risk of contracting COVID-19	No	760 (25%)	618 (21%)	805 (27%)	626 (21%)	**ref**	**ref**
	Yes	2240 (75%)	2382 (79%)	2195 (73%)	2374 (79%)	1.391 (1.234–1.567)***	1.380 (1.180 – 1.612)***
Being afraid of transmitting COVID-19 to others	No	964 (32%)	847 (28%)	1000 (33%)	882 (29%)	ref	ref
	Yes	2036 (68%)	2153 (72%)	2000 (67%)	2118 (71%)	1.201 (1.076–1.339)**	0.933 (0.807–1.080)
Wearing facemask	No	215 (7%)	171 (6%)	186 (6%)	166 (6%)	ref	ref
	Yes	2785 (93%)	2829 (94%)	2814 (94%)	2834 (94%)	1.128 (0.910–1.400)	1.008 (0.789–1.288)

*p < 0.05; **p < 0.01; ***p < 0.001

Based on the cumulative number of COVID-19 cases per 100,000 population as of 2 May 2020, the 20 studied prefectures were divided into low (bottom 10) and high (top 10) areas

*AOR* adjusted odds ratio, *OR* odds ratio, *CI* confidence interval; ref: reference category

^a^Avoiding all 3 Cs means that avoiding ‘closed spaces with poor ventilation’, ‘crowded places’ and ‘close contact setting’, such as close-range conversations

^b^‘Avoiding all 3 Cs’, includes the broken down three behaviours. Therefore, this variable was excluded from the multivariate binominal analysis

## Discussion

According to the definitions established by the World Health Organization and the United Nations, when more than 21% of a population is aged ≥ 65 years, the society is called a ‘super-aged society’ [11]. In Japan, the proportion reached 28% in 2019 [10]. This study revealed the existence of a significant negative correlation between ‘CASES per 100,000 on 02/05/2020’ and the proportion of the population aged ≥ 65 years in Japan. The correlation coefficient with the cumulative COVID-19 cases per 100,000 population on 2 May 2020 was − 0.701 (*p* < 0.001). In the 10 prefectures with low COVID-19 incidence, the median proportion of the population aged ≥ 65 years was 30%, and the proportions in 4 of the10 prefectures exceeded 30% (Table 1, Fig. 1). Furthermore, in the same area, the proportion of younger people (18–29 years) was extremely low, with a median of only 9.8%. Their proportion in nine of the 10 prefectures was less than 10%. The proportion above makes it clear that there was a polarization of population age structures between the low- and high-incidence areas.

Besides population density, the polarised nature of the COVID-19 pandemic in Japan was associated with age specific people’s behaviours and perceived risk of contracting COVID-19. As shown in Fig. 3 and Table 3, ‘age’ influenced people’s behaviours and perceptions, except regarding ‘wearing facemasks’. Older adults are less likely to commute by public transportation and to visit places where alcohol is served than younger people. In addition, they are more likely to wash hands and to avoid the 3 Cs, even though their perceived risk of contracting COVID-19 was relatively lower than that among younger people. In contrast, the proportion of individuals from the population group of aged 18–29 years who avoided all the 3 Cs was < 30% in both areas, despite the higher perceived risk of COVID-19. The proportion was far less than the target set by the Japanese government of 70% to 80% [4].

The variables of the 3 Cs should be discussed along with the means of commute and behaviours linked with alcohol intake. In low-COVID-19-incidence area, public transportation was not convenient because of insufficient infrastructure. Therefore, it is common for residents in such areas to use their private cars to commute. In contrast to the low-incidence area, high-incidence area had developed public transportation networks commonly used for commuting. The characteristics of public transportation in high-incidence area are well described in consideration of the 3 Cs, as Iwasaki et al. [5] mentioned that social distancing was negligible during rush hours in trains and buses. Therefore, ‘commuting by public transportation’ was positively associated with high-incidence area, whereas ‘commuting by private automobile’ was negatively associated with the area. These two variables strongly influence the incidence of COVID-19, as shown in Table 4. In addition, ‘non-scheduled visit to drinking places’ was positively associated with high-incidence area, while ‘going eat’ was insignificantly associated with the high-incidence area. Generally, in the drinking spots, people may have difficulties to avoid 3 Cs, particularly avoiding close-contact setting such as close-range conversations. Also, under an influence of alcohol, people are less likely to practice preventive measures due to impaired normal judgements by the alcohol intake [12].

Regarding the perceived susceptibility to severe acute respiratory syndrome coronavirus 2 (SARS-CoV-2), the proportion of individuals who perceived was higher in the high-incidence area than in the low-incidence area. The government of Japan has aggressively promoted advocacy campaigns to prevent COVID-19 since February 2020 [3, 9, 13], and respondents residing in high-incidence areas seem more stimulated with a daily increase in new COVID-19 cases [14, 15]. However, the higher perceived susceptibility does not motivate the younger population to practice preventive measures as aforementioned.

Older adults are considered vulnerable to COVID-19 in terms of susceptibility and severity once infected by SARS-CoV-2 [16]. This does not necessarily mean that they are the main source of virus transmission, because once an older person gets infected, his/her mobility will be lowered due to an increased risk of severe illness and/or death [16, 17]. The source of transmission, except in clinical settings, might be much younger individuals. Compared with the low-incidence area, the high-incidence area had more individuals aged 18–29 years. Younger people are more likely to be asymptomatic and transmit SARS-CoV-2 due to their numerous social activities [18–21]. Thus, it may not be entirely wrong to conclude that younger people are the source of COVID-19 transmission. Additionally, in Japan, the observed gap between low and high COVID-19 incidence was associated with the population age structure as discussed earlier. The results of this study suggest that age-specific strategies should be established, and an approach to fill the gaps in the understanding of preventive measures should be subsequently developed.

This study has some limitations. The sample size was not decided by population-based proportionate sampling; instead, it was decided with the intention to maximise the number of participating individuals aged ≥ 60 years and 18–29 years. Therefore, each prefecture sample was weighted using age-specific proportions of the population by sex and size. As only 300 samples were collected from each prefecture, it was challenging to analyse each prefecture situation; therefore, all analyses were carried out using aggregated data from low- and high-incidence areas and younger and older age groups. This study covers up to 87-year-olds and 89-year-olds in low- and high-incidence areas, respectively. The author believes that many older people who could not be included in this study are less active due to an age related state of frailty; thus, their ability to prevent COVID-19 should be much less than that of the study respondents except the cases who contracted COVID-19 as a nosocomial infection while staying at day-care centres or hospitals. Therefore, it is necessary to keep it in mind when the study implications are interpreted. However, it is very challenging to access older individuals for interviews in Japan.

## Conclusion

There were differences in preventive behaviours and risk-related perceptions between young and older adults. Commuting measures were strongly associated with COVID-19 incidence, and they seemed related to being in crowded and closed spaces with poor ventilation along with visiting drinking spots. Effective preventive measures for COVID-19 transmission, particularly advocacy campaign, can be developed by understanding the characteristics of populated areas, such as public transportation infrastructure and younger people’s movements and behaviours in relation to the population age structure to contain the current epidemic and protect the most vulnerable elderly people. Finally, in Japan, the rates of wearing facemasks in both low- and high-incidence areas were very high (94% in both areas), with no statistically significant differences between the rates. This study could not test its efficacy in containing COVID-19, although, several studies have shown its efficacy [22–24].

I hope that the variables collected through this study such as the proportions of those who adhere to the preventive practice and who perceived risk might be useful for establishing mathematical models to project the direction and magnitude of COVID-19 infection in Japan as realistic parameters.

## Data Availability

The data sets are written in Japanese. They are available upon reasonable request

## Abbreviations

3 Cs: Closed spaces with poor ventilation, crowded places and close contact setting, such as close-range conversations
AOR: Adjusted odds ratio
CI: Confidence interval
COVID-19: Coronavirus disease
IQR: Interquartile range
OR: Odds ratio
SARS-CoV-2: Severe acute respiratory syndrome coronavirus 2
