# Correction to: Polarized nature of the COVID-19 pandemic in Japan: associations with population age structure and behaviours

**DOI:** 10.1186/s41182-021-00350-y

**Published:** 2021-07-29

**Authors:** Junko Okumura

**Affiliations:** 1 Institute of Tropical Medicine, Nagasaki, Japan; 2 School of Tropical Medicine and Global Health, Nagasaki University, 1-12-4 Sakamoto, Nagasaki, Nagasaki 852-8523 Japan

**Correction to: Trop Med Health 49, 38 (2021)**

**https://doi.org/10.1186/s41182-021-00324-0**

Following publication of the original article [1], the authors identified an error in Fig. 1. The wrong part is the location of the red-line to indicate the 30% line. The correct figure is given below.

**Fig. 1:**
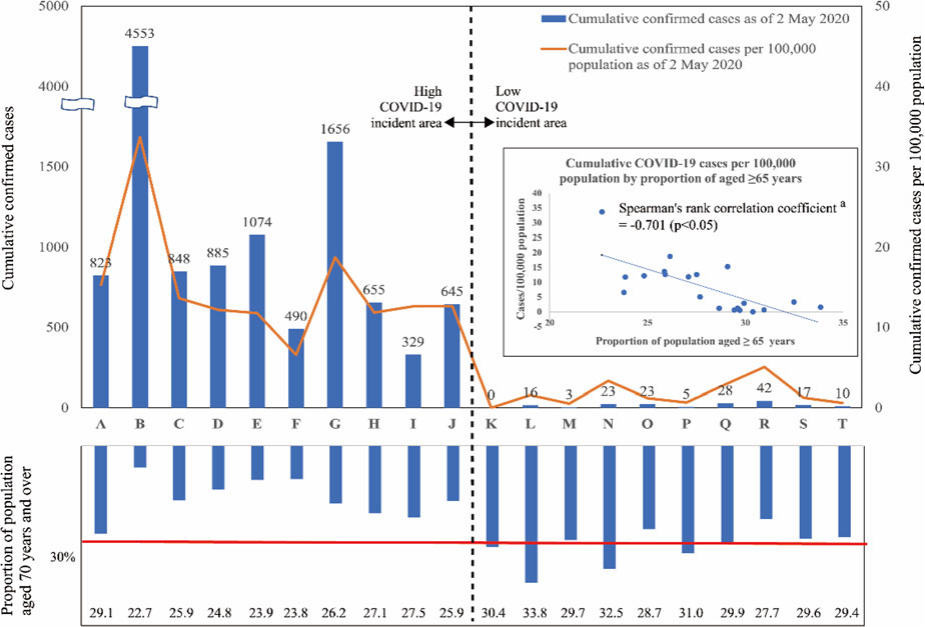
Cumulative COVID-19 cases, cases per 100,000 population and proportion of population aged ≥65 years. ^a^ Due to non-normal distribution of data, Spearman’s rank correlation test was adopted. Cumulative COVID-19 cases and cases per 100,000 population are calculated based on reported cases as of 2 May 2020

The original article [1] has been corrected.
